# Geography and prevalence of rickettsial infections in Northern Tamil Nadu, India: a cross-sectional study

**DOI:** 10.1038/s41598-022-21191-7

**Published:** 2022-12-02

**Authors:** Solomon D’Cruz, Susmitha Karunasree Perumalla, Jayaraman Yuvaraj, John Antony Jude Prakash

**Affiliations:** 1 Department of Clinical Microbiology, Christian Medical College, Vellore, Tamil Nadu India; 2 Health Systems Research, ICMR-National Institute of Epidemiology (NIE), Chennai, Tamil Nadu India

**Keywords:** Epidemiology, Bacterial infection

## Abstract

Rickettsial infections and Q fever are a common cause of acute febrile illness globally. Data on the role of climate and altitude on the prevalence of these infections in lacking from Southern India. In this study, we determined the sero-prevalence of scrub typhus (ST), spotted fever (SF), murine typhus (MT) and Q Fever (QF) in 8 eight geographical regions of North Tamil Nadu by detecting IgG antibodies using ELISA. Totally we tested 2565 people from 86 localities. Among the 27.3% positives, approximately 5% were IgG positive for two or more infections. Sero-prevalence to rickettsioses and Q fever was highest for individuals from rural areas and increased with age (> 30 years). Those in the Nilgiris highlands (wetter and cooler) and Erode, which has the most land under irrigation, demonstrated the least exposure to rickettsioses and Q fever. Lowland plains (AOR: 8.4–22.9; 95% CI 3.1–55.3) and highland areas up to 1000 m (AOR: 6.1–10.3; 95% CI 2.4–23.9) showed the highest risk of exposure to scrub typhus. For spotted fever, the risk of exposure was highest in Jawadhi (AOR:10.8; 95% CI 2.6–44.3) and Kalrayan (AOR:16.6; 95% CI 4.1–66.2). Q fever positivity was most likely to be encountered in Salem (AOR: 5.60; 95% CI 1.01–31.08) and Kalrayan hills (AOR:12.3; 95% CI 2.9–51.6). Murine typhus risk was significant only in Tiruvannamalai (AOR:24.2; 95% CI 3.3–178.6). Our study suggests that prevalence of rickettsial infections and Q fever is low in areas which receive rainfall of ≥ 150 cm/year, with average minimum and maximum temperatures between 15 and 25 °C and elevation in excess of 2000 m. It is also less in well irrigated lowlands with dry climate. These preliminary findings need confirmation by active surveillance in these areas.

## Introduction

Rickettsial diseases have emerged as one of the major public health threat to the mankind. The agents causing these infections belong to the Order *Rickettsiales* which comprises of the family *Anaplasmataceae* and *Rickettsiaceae*. Of these rickettsioses are caused by bacteria belonging to the genus *Rickettsia*, while scrub typhus is due to *Orientia *spp.^1^ and Q Fever by genera *Coxiella*^2^. The aforementioned rickettsial infections are vector borne zoonotic diseases caused by various group of obligate intracellular bacteria^3^ transmitted to humans through the bite of infected ticks, mites, fleas and lice^4^. In contrast, Q Fever occurs most often due to inhalation of the infected particles from infected mammals^5^. Rickettsioses are very challenging to diagnose and has a high fatality rate of 9–70% among the untreated patients^6–10^ and are a global public health threat^6,11,12^. Scrub typhus, once thought to be endemic only in the “tsutsugamushi triangle”, is now being reported from places like Africa, France, Middle East, South America. This is a clear indication that scrub typhus is no longer restricted to the “tsutsugamushi triangle”^9^.

The sero-prevalence of scrub typhus (ST), spotted fever (SF), murine typhus (MT) and Q Fever (QF) in Bhutan was 22.6%, 15.7%, 3.5% and 6.9% in 2017^13^. In Northeast India, the sero-prevalence was found to be 30.8% for ST, 13.8% for SF and 4.2% for MT^14^. The sero-prevalence of ST, SF and MT in South-India was found to be 20.4%, 10.4%, and 5.4% respectively^15^. Not many studies have been performed to determine the sero-prevalence of Q Fever among humans in India^16,17^.

Sero-prevalence of Scrub typhus was found high during the winter and rainy season in Nilgiris^18^ and Mizoram^19^. Scrub typhus is common in plains^15^ and spotted fever is more prevalent in the hilly regions^20^. The geographic variations like change of altitude, temperature, rainfall, humidity and the effects of the same on the prevalence of murine typhus and Q fever still remain unexplored. In this study we have conducted a sero-prevalence study across different terrains for better understanding the role of geography on the occurrence of rickettsial infections and Q fever.

## Methodology

### Study type and study setting

A community based cross-sectional study was undertaken in 5 districts of North Tamil Nadu namely Erode, Nilgiris, Salem, Tiruvannamalai and Vellore from September 2017 to January 2020. These five districts of Tamil Nadu vary geographically across and within themselves.

The major occupation of people in all 5 districts is agriculture with paddy being the major crop grown in Erode and Vellore, millets and other cereals in Salem, Tiruvannamalai and tea plantation in Nilgiris district. Erode district has the least forest cover with 14.25%, followed by Salem, Tiruvannamalai and Vellore with 22%, 24.20% and 27.94% respectively. On the contrary, Nilgiris has the maximum forest coverage with 56% of forest in the entire district. In terms of climatic zones, Nilgiris falls in temperate climatic zone and tropical climate is seen in Salem, Tiruvannamalai, Erode and Vellore (https://www.tn.gov.in/deptst/agriculture.pdf)^21^.

Vellore is densely populated with 648 people per km^2^. followed by Salem, Tiruvannamalai and Erode with 573, 399 and 391 respectively. Nilgiris is the sparsely populated district with only 289 people per km^2^ (https://censusindia.gov.in/2011census/dchb/Tamilnadu.html)^22^.

The five surveyed districts were divided further into 8 areas for analysis (Erode, Nilgiris, Jawadhi, Kalrayan, Palamathi, Tiruvannamalai and Vellore). This is to distinguish the geographical differences that exist even within the selected district itself. The district of Salem was divided into Salem plain and the Kalrayan hills which is located 600–1100 m above the sea level. Tiruvannamalai district was divided into plains of Tiruvannamalai and Jawadhi hills (only the part which is in Tiruvannamalai district). Vellore was divided into Vellore plains, Jawadhi hills (part of Vellore district) and Palamathi hills which have an elevation 600–1000 m and 300–600 m above the sea level respectively (https://en-ca.topographic-map.com/maps/lggf/Tamil-Nadu/)^23^. Table 1 describes the average elevation of the study areas.

**Table 1:** Climate variables and sero-prevalence of rickettsial and Q fever in the study areas.

Regions surveyed	Average elevation (range) in metres^a^	Rainfall (cm)^b^	Max temp in °C (range)^b^	Min temp in °C (range)^b^	Humidity % (range)^b^	ST (%)	SF (%)	MT (%)	QF (%)
Jawadhi	598 (500–773)	99	34.1 (30–39)	24.3 (21–27)	71 (61–82)	14.6	20.5	0	1
Palamathi	285 (230–413)	99	34.1 (30–39)	24.3 (21–27)	71 (61–82)	12.3	6.8	0	0.7
Kalrayan	795 (673–982)	100	29.8 (26.2–34)	20.4 (16.8–23.1)	64 (45–75)	16	29.9	2.4	11.5
Nilgiris	2046 (1933–2229)	151	21 (19.7–23.4)	13.2 (10.6–15)	75 (57–85)	2	5.2	2	4.3
Salem	251 (153–309)	70	35.1 (30–39)	24.2 (21–27)	66 (57–79)	10.5	1.7	0.6	11.6
Vellore	217 (206–246)	94	32.4 (27.2–37.4)	22.2 (16.9–26.2)	63 (52–77)	21	5.2	2.4	6.7
Tiruvannamalai	180 (147–216)	81	32.9 (27.9–37.4)	22.9 (18–26.5)	63 (53–76)	26.4	8.2	13.4	1.7
Erode	207 (157–275)	80	32.8 (29.1–37.4)	22.8 (19.9–25.7)	61 (42–72)	2.2	0	2.2	6.7

^a^Of the areas where sero-surveillance was conducted in this study.

^b^Data for 2017 (Jan–Dec).

### Sample size

Assuming a sero-prevalence (p) of rickettsioses of 25% among the 12.8 million population according to the 2011 census of the 5 districts surveyed (https://www.census2011.co.in/census/state/districtlist/tamil+nadu.html)^24^. The precision was set at 3% and the sample size was calculated for a 95% confidence interval and a 5% of margin of error. Using a design effect of 3, the calculated sample size was 2399 for the study as calculated by the OpenEpi Software^25^.

### Sampling technique

The study included 86 clusters which were selected from the 5 districts according to the feasibility of the investigator in terms of obtaining permission from the local body, people consenting and accessibility of the locality. In each cluster, 30 households were selected by using systematic sampling technique. The sampling interval was determined by the number of households (https://www.census2011.co.in/census/state/districtlist/tamil+nadu.html)^24^ in that particular locality/village and dividing it by 30. The base point of the survey was started from the south-east end of each village/locality moving Northwards in an east–west pattern (zig-zag manner). In case of locked house or denied consent, the very next house was surveyed to complete the sample size. One participant from each household was randomly selected to participate in the study. Selected subjects were residents who had stayed continuously in the locality for ≥ 2 years, who reported no history of fever for the past three months, > 15 years of age of either sex were included. Participants who were not willing to participate in the study, who were immunosuppressed or had fever at the time of serum sampling were excluded. Informed consent was obtained from participants or their legal guardians prior to recruitment into the study.

Socio-demographic data and 4 ml blood was collected in BD Vacutainer Clot Activator Tube (BD, Plymouth, UK) from all the study participants. The serum was separated by centrifugation and tested for detection of IgG antibodies to Scrub Typhus, Spotted fever, Murine Typhus and Q fever at a dilution of 1:100. IgG antibodies to *O. tsutsugamushi* were detected using the Scrub Typhus Detect IgG ELISA system (InBios International Inc., Seattle, WA). While IgG antibodies to murine typhus and spotted fever were detected by *R. typhi* IgG ELISA (Fuller Laboratory, Fullerton, CA) and the Rickettsia conorii ELISA IgG/IgM (Vircell, Granada, Spain). As described previously, a serum sample was considered to be positive if the OD was ≥ 1.5 for any of these infections. This was based on the bimodal peak observed in earlier scrub typhus IgG ELISA studies, which was extrapolated to spotted fever and murine typhus^15^. Q Fever Phase 2 IgG antibodies were detected using the NovaLisa *Coxiella burnetii* (Q-fever) Phase 2 IgG ELISA (NovaTec Immundiagnostica GmbH, Dietzenbach, Germany). All the assays were performed with appropriate controls on an automated ELISA workstation (Euroimmun Analyzer I, Euroimmun AG, Lubeck, Germany). The OD cut-off for Q Fever was set at 0.6 and above since the mean plus 2 SD was just above 0.6 (Data not shown). The outcome of the study is the prevalence of sero-positivity of Scrub Typhus, Spotted fever, Murine typhus and Q Fever and its association with various geographic factors. IgG ELISA was chosen to define exposure as IgG antibodies persist for a long time and indicate past exposure, automated ELISA was used as we screened large number of samples for 4 different agents. The methods performed to assess the various parameters, conformed to relevant guidelines and regulations (laboratory is ISO15189:2012 compliant and accredited). The exposure variables that were measured in this study are age, gender, place of residence (urban/rural), elevation of the area, presence of peri-forest and the locality. Elevation profile of each locality was obtained from Bharat Maps (https://bharatmaps.gov.in/newversion/map.aspx)^26^. The average, minimum and maximum temperatures, humidity and rainfall were captured from Time and Date website (https://www.timeanddate.com/)^27^. These are as given in Table 1.

### Statistical methods

The data was entered using Epidata 3.1 version (Jens M. Lauritsen, Odense, Denmark) and analyzed using IBM SPSS Statistics for Windows, Version 21.0 (IBM Corp, Armonk, NY, USA). The continuous variables are expressed in mean and standard deviation (SD) for normal distribution, median and inter-quartile range (IQR) for skewed distribution. The categorical variables are expressed in frequencies and percentages. The association between the categorical variables is analyzed using Chi-Square test and the unadjusted odds ratio (OR) was reported as the strength of association with 95% Confidence Interval (95% CI). Logistic regression statistics was done to adjust for confounders and adjusted odds ratio (AOR) was presented with 95% CI. The level of significance of p value was set at < 0.05.

The study protocol was approved by the Institutional Review Board (IRB) and Ethics committee of the Christian Medical College, Vellore, India (vide IRB Min. No. 9369 dated 25th March 2015). The study was conducted as per the norms prescribed in the Declaration of Helsinki.

### Ethics approval statement

The study was approved by the institutional review board (IRB) and ethics committee (EC) of the Christian Medical College, Vellore, India (vide IRB Min. No. 9369 dated 25th March2015).

## Results

### Demographic characteristics

The total number of participants recruited for the study was 2565 from 86 localities/villages of the 5 districts surveyed. Out of all study participants 60% were female, 31.5% belongs to the age group of 31–45 years. The people living in peri-forested area are 43.2% and 69.1% of the population are living in the rural area. Participants from plains of Vellore were the largest group (Table 2). Proportion of positivity with relation to samples tested are given in Supplementary Datasets 1 and 2.

**Table 2:** Demographic features of study subjects.

(n = 2565)		Frequency	Percentage
Sex	Male	1025	40
	Female	1540	60
Age group	16–30 years	641	25
	31–45 years	807	31.5
	46–60 years	692	27
	> 60 years	425	16.6
Place of residence	Urban	793	30.9
	Rural	1772	69.1
Peri-forested	Yes	1108	43.2
	No	1457	56.8
Survey areas	Erode	406	15.8
	Jawadhi Hills	205	8
	Nilgiris	345	13.5
	Palamathi Hills	146	5.7
	Kalrayan Hills	331	12.9
	Salem	181	7.1
	Tiruvannamalai	417	16.3
	Vellore	534	20.8

### Sero-prevalence of rickettsial and Q fever

The sero-prevalence of scrub typhus, spotted fever, murine typhus and Q fever in the eight regions surveyed is as given in Table 1.

**Figure 1:**
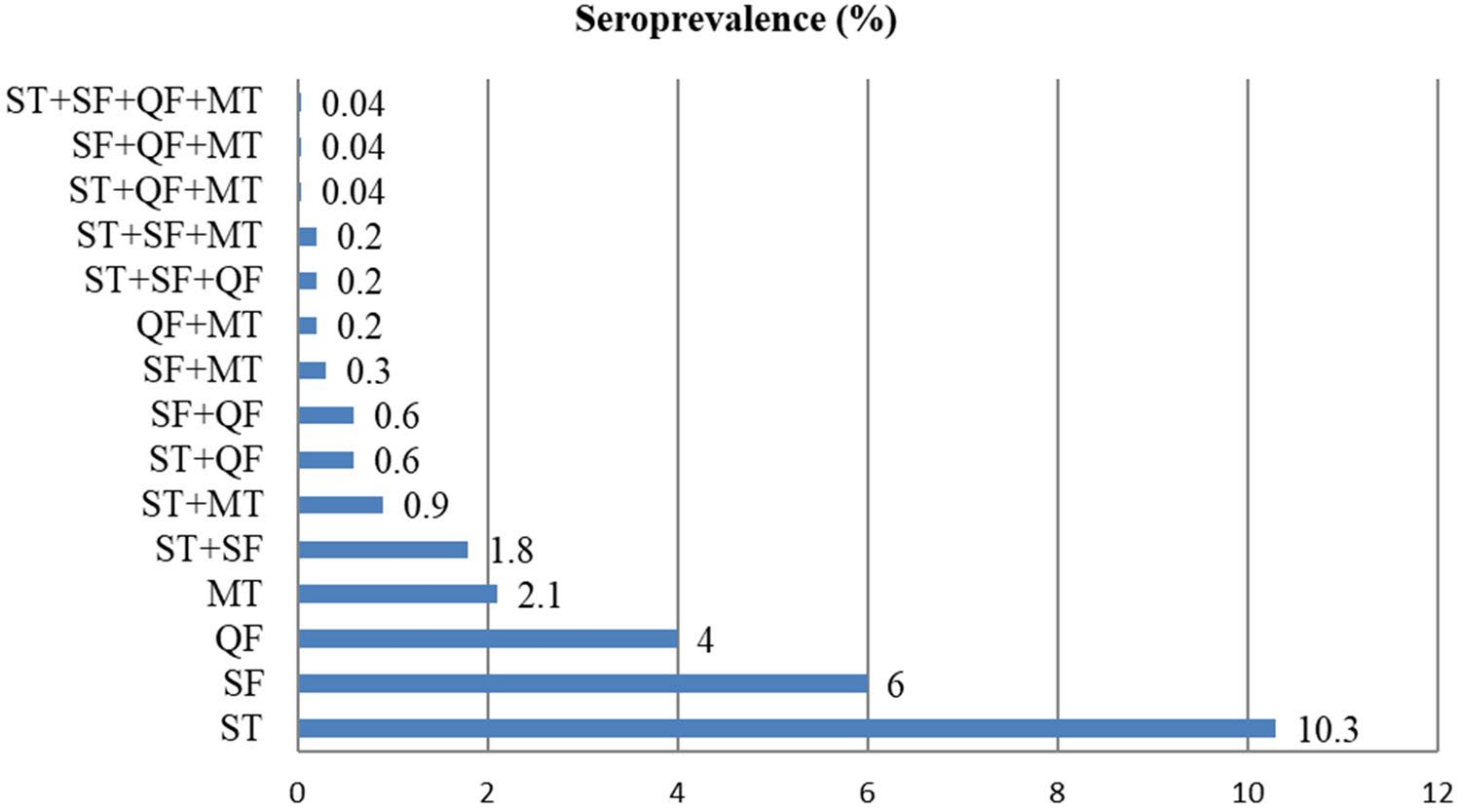
Sero-prevalence of rickettsioses in the regions studied.

The overall sero-prevalence for any one of the Rickettsial infection in the study population is 22.4%. The proportion of people sero-positive for any 2 rickettsial infection is 4.4%, and 0.5% for any 3 and 0.04% for all 4 rickettsial infections. Overall positivity including single and multiple rickettsial infection is 27.3%. Details are as show in the Fig. 1.

The highest prevalence of ST was seen in plains of Tiruvannamalai and plains of Vellore with 26.4% and 21% respectively and the lowest prevalence was seen in Nilgiris and Erode with 2% and 2.2% respectively. SF was high in Kalrayan hills and Jawadhi hills with a sero-prevalence of 29.9% and 20.5% respectively and low in plains of Salem with 1.7%. There was no case of SF in Erode. High prevalence of MT was observed in plains of Tiruvannamalai with 13.4% and low in plains of Salem with 0.6%. There was no case of MT in Jawadhi hills. Plains of Salem reports a high prevalence of QF with 11.6% of sero-prevalence and the lowest prevalence of 0.7% was observed in Palamathi hills (Supplementary Datasets 3 and 4).

### Univariate analysis

Details of the parameters used for univariate analysis and the results obtained are as given in Supplementary Dataset 4. Univariate analysis suggested females were more likely to be sero-positive for ST and MT, whereas such trend was not observed with SF and QF. The significantly associated risk factors of ST and SF in univariate analysis were age and residing at rural areas (refer Suppl Dataset 4). Age proved to be a strong associated risk factor for sero-positivity of ST with a significant increase in risk for all aged above 30 years. People residing at rural and non peri-forested areas had an increased association with ST when compared to people living in urban and peri-forested areas. Plains of Tiruvannamalai and Vellore found to be the hotspots of Scrub Typhus with a significantly increased risk when compared to the other survey areas. For SF, the risk factors which were significantly associated were people aged 46–60 years, living in rural and peri-forested areas. When compared to plains of Salem, people living in Jawadhi, Palamathi, Kalrayan and plains of Tiruvannamalai were at high risk for SF. People from areas which are not peri-forested areas and plains of Tiruvannamalai were the only significantly associated risk factors for MT when compared to peri-forested areas and plains of Salem. For Q fever, people aged 31–45 years and > 60 years are at high risk in univariate analysis. Compared to the Palamathi hills, people living in Kalrayan, plains of Salem, Erode and plains of Vellore were at high risk for Q Fever (refer Suppl Dataset 4).

### Multivariate analysis

Gender, age group, place of residence, peri-forest and survey areas were found to be significant by univariate analysis for one or more of the four infections studied. Table 2 depicts the multivariate analysis results. In multivariate analysis, females were found to be significantly associated with MT (AOR: 2.09, 1.26–3.46). The associated risk of age for ST has increased significantly as the age of the participants increased from 26 to above 65 years. In contrast, the people aged from 46 to 65 years had a significant increase in the association of SF. Age had no bearing for Q Fever risk whereas significant risk for murine typhus was observed among those aged 46–55 years (AOR: 2.70, 1.15–6.33) and above 65 years (AOR: 3.27, 1.18–9.06). The people residing in rural areas had a significant associated risk of being sero-positive for ST (AOR: 2.39, 1.73–3.30), SF (AOR: 2.46, 1.41–4.31) and MT (AOR: 1.77, 1.07–2.91) when compared to people residing in urban areas. When compared to Nilgiris, all the other survey areas had increased odds of being sero-positive for Scrub typhus except Erode with the maximum risk for people residing in plains of Tiruvannamalai (AOR: 24.10, 9.97–58.27). People residing in Kalrayan hills had a high risk of being sero-positive for SF, when compared to plains of Salem. The other survey areas which had significant association with SF are Jawadhi and plains of Tiruvannamalai. For Q Fever, the highest risk was found in Kalrayan hills (AOR 12.51, 2.98–52.56), while. plains of Tiruvannamalai (AOR: 26.94, 3.63–200.23) had a significant association with the sero-positivity for MT when compared to other areas (Table 3).

**Table 3:** Risk factors for rickettsial infection.

Risk factors (n = 2565)		Scrub typhus		Spotted fever		Q fever		Murine typhus	
		AOR (95% CI)	p value	AOR (95% CI)	p value	AOR (95% CI)	p value	AOR (95% CI)	p value
Sex	Male	Reference							
	Female	1.06 (0.82–1.36)	0.655	0.82 (0.61–1.10)	0.192	0.83 (0.58–1.18)	0.295	**2.09 (1.26–3.46)**	**0.004**
Age group (years)	15–25	Reference							
	26–35	1.16 (0.70–1.92)	0.567	1.67 (0.95–2.92)	0.073	1.23 (0.62–2.45)	0.547	2.19 (0.94–5.11)	0.070
	36–45	**2.55 (1.60–4.08)**	** < 0.001**	1.39 (0.78–2.47)	0.263	1.74 (0.92–3.30)	0.089	1.94 (0.82–4.57)	0.131
	46–55	**2.89 (1.78–4.67)**	** < 0.001**	2.39 (1.37–4.19)	**0.002**	1.24 (0.62–2.49)	0.544	**2.70 (1.15–6.33)**	**0.023**
	56–65	**3.88 (2.40–6.30)**	** < 0.001**	2.10 (1.18–3.73)	**0.012**	1.55 (0.79–3.07)	0.204	1.37 (0.53–3.55)	0.523
	> 65 years	**3.39 (1.93–5.95)**	** < 0.001**	1.92 (0.95–3.87)	0.068	2.03 (0.98–4.22)	0.058	**3.27 (1.18–9.06)**	**0.023**
Place of residence	Rural	**2.39 (1.73–3.30)**	** < 0.001**	**2.46 (1.41–4.31)**	**0.002**	Reference		1.77 (1.07–2.91)	0.026
	Urban	Reference				1.38 (0.86–2.21)	0.179	Reference	
Peri-forested	No	1.39 (0.90–2.13)	0.134	Reference		1.63 (0.66–4.00)	0.292	1.47 (0.59–3.64)	0.406
	Yes	Reference		0.71 (0.35–1.43)	0.336	Reference			
Area surveyed	Erode	1.13 (0.39–3.28)	0.830	NA	NA	3.59 (0.68–19.01)	0.132	3.72 (0.46–30.25)	0.218
	Jawadhi	**10.38 (4.43–24.29)**	** < 0.001**	**10.85 (2.65–44.48)**	**0.001**	Reference		NA	NA
	Kalrayan	**9.62 (4.28–21.59)**	** < 0.001**	**16.90 (4.21–67.86)**	** < 0.001**	**12.51 (2.98–52.56)**	**0.001**	4.59 (0.47–44.94)	0.191
	Palamathi	**6.11 (2.36–15.82)**	** < 0.001**	2.39 (0.60–9.49)	0.218	0.47 (0.04–5.70)	0.551	NA	NA
	Nilgiris	Reference		2.24 (0.53–9.49)	0.274	4.18 (0.94–18.57)	0.060	3.71 (0.37–36.77)	0.263
	Salem	**8.69 (3.21–23.55)**	** < 0.001**	Reference		5.60 (1.01–31.10)	0.049	Reference	
	TVmalai	**24.10 (9.97–58.27)**	** < 0.001**	**3.97 (1.16–13.52)**	**0.028**	0.93 (0.16–5.56)	0.935	**26.94 (3.63–200.23)**	**0.001**
	Vellore	**15.11 (6.46–35.34)**	** < 0.001**	2.31 (0.66–8.01)	0.189	4.12 (0.81–20.89)	0.088	3.78 (0.48–29.90)	0.207

Significant values are in bold.

**Figure 2:**
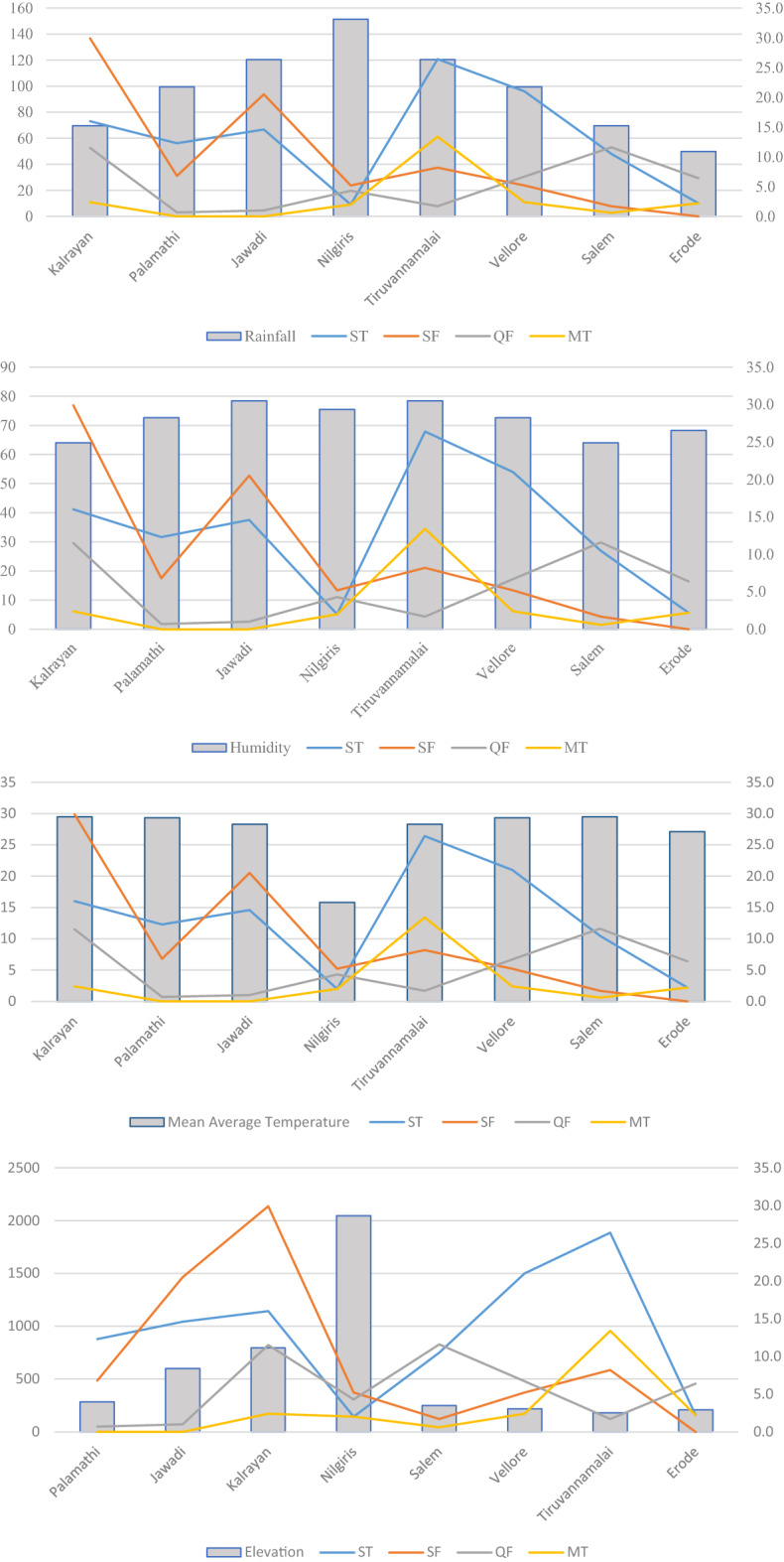
Geographic variables and prevalence of rickettsioses in North Tamil Nadu (**A**) Rainfall per annum in cm. (**B**) Average daily humidity in percent. (**C**) Mean average daily temperature in celsius. (**D**) Mean elevation in metres.

The elevation (in metres), total rainfall (in cm), temperature (in °C), Humidity (in %) and sero-prevalence of the four rickettsial infections in percentage with range in parenthesis as applicable is given Table 1. Figure 2A–D give the sero-prevalence of scrub typhus (ST), spotted fever (SF), murine typhus (MT) and Q fever (QF) across the 8 regions and their relation to temperature, humidity, rainfall and elevation.

## Discussion

This study aimed to find the out the sero-prevalence of ST, SF, MT, QF and its variation across various geographical areas of Northern Tamil Nadu. ST was found to be the most common of all the rickettsial infections observed in this study.

The overall sero-prevalence of rickettsioses (ST, SF, MT and Q Fever) was 32.5%, this is lower than the 48.7% reported by Tshokey et al.^13^ in Bhutan for these infections. In 4.76% of our subjects we observed sero-prevalence for two or more infections, whereas Tshokey et al. 2017 reported a rather high 11.15% using immunofluorescence^13^. In a study, performed on 99 North Korean refugees, Um et al., reported that 2% of the samples were positive for all three diseases tested, scrub typhus, spotted fever and murine typhus, while 8.1% were positive for both SF and MT^28^. This could be due to cross reactions or co-infections or past exposure as the risk factors for acquisition of these diseases are the same^29^.

The observed sero-prevalence of ST and SF in this study is less than the sero-prevalence reported in Bhutan (ST—22.6%, SF—15.7%) by Tshokey et al. in 2017^13^ and in Northeast India (ST—30.8% and SF—13.8%) by Khan et al. in 2016^14^. On the contrary, the finding of this study for MT was found to be almost similar to the sero-prevalence reported in Bhutan (MT—3.5%)^13^, Spain (MT—3.8%) in 2006^30^ and in Northeast India (MT—4.2%) in 2016^14^. The sero-prevalence of Q Fever is slightly lower than the reported sero-prevalence in Bhutan (QF—6.9%) by Tshokey et al.^13^ and in Sao Tome Island (QF—6.7%) by Hsi et al.^31^.

There was no influence of gender found in ST, SF and QF in this study which is in-line with other studies conducted in China^32^ for ST, Londono et al.^33^ in 2016 for SF and Mostafavi et al.^34^ for Q Fever in 2018. Females were found to be more likely to be sero-positive for murine typhus (MT) in our study, which is in contrast with other studies which report no significant association of gender and MT in Peru^35^ and Korea^36^.

Age was found to be an important risk factor for ST, SF and MT, with the increase of age there was an increased risk for ST and SF in our study. The same trend was reported in Vientiane city^37^, Bhutan^13^ for ST and for SF in Gorakhpur^38^ and Greece^39^ and in Brazil by da Costa et al^40^. An increasing trend for Q fever sero-prevalence was observed in the age group of 26 years to above 65 years of age which is in-line with the observations of Dupont et al. in France^41^. From, Minas Gerais, Brazil, da Costa et al., reported that age > 40 years correlated with increased Q fever sero-prevalence^40^. In this study, age was significantly associated with MT which is in consonance with the study done in Peru where the age was found to be a significantly associated with the sero-positivity of MT^35^.

We observed a higher sero-prevalence of ST in rural areas (15.3%) and lower in urban areas (10.8%). A similar pattern was reported by Vikram et al. in 2020 with the sero-prevalence of 25% among rural and 18.1% among urban population^42^. Residing in the rural areas was found to be a potential risk factor for spotted fever and murine typhus as for scrub typhus in this study. The same was observed for ST in Rajasthan by Bithu et al. in 2014^43^, Daniel et al.^39^ for SF in Greece, Forshey et al. in Peru in 2010^35^ and Sri Lanka in 2012^29^ and for MT by Chang et al. 2018 in Korea^36^ and by Hidalgo et al. in Colombia^44^. Weitzel et al. observed that those residing in rural areas in Chile, had a higher sero-prevalence of scrub typhus, murine typhus and spotted fever^45^. It is to be noted that residents of urban areas where more likely to be QF positive in univariate analysis, but not in multivariate analysis, in the present study.

In South America, murine typhus sero-prevalence of around 1% has been reported from Argentina, Peru, Brazil and Chile, whereas a high prevalence of 25.2% has been documented from Caldas, Colombia. SFGR prevalence ranging from 5% in Chile, 6–14% in Peru and 28% from Argentina has been reported^45^.

The sero-prevalence of all rickettsial infections including Q Fever differs significantly across various surveyed area in this study. A similar trend was observed for ST and SF in Bhutan^13^, for SF in Greece^39^ and Reunion island^46^, for MT in Korea^36^ in Reunion island^46^ and QF in Jordan^47^. ST is the common rickettsial infection observed in the plains of Vellore and Tiruvannamalai and uncommon in hilly Nilgiris and well irrigated intensely cultivated lands of Erode. Philip et al.^18^ reported that only 6.07% had significant IgM antibodies for ST amongst the 214 AFI cases tested in the Nilgiris. This might be due to the unfavorable environmental conditions, like low temperature and high altitude conditions for the mite to survive^13^. Wangramsimakul et al. have opined that scrub typhus incidence was lower in areas under intense cultivation due to availability of irrigation facilities^48^. A meta-anlysis by Shah et al., demonstrated that vector borne illnesses like scrub typhus were far more likely to occur in areas with abundant rubber and palm oil plantations rather than irrigated rice paddies^49^. This is consistent with our finding of low frequency of rickettsioses in Erode which has large areas of well irrigated intensively cultivated cropland. Further as scrub typhus is a chigger borne illness, chigger activity which is closely related to temperature and humidity and rainfall will also play a major role in sero-prevalence of this disease^50,51^.

SF seems to be more common among the hilly areas with elevation below 1000 MSL like Kalrayan and Jawadhi hills. This is supported by our preliminary results published in 2020^15^ and Kularatne et al. in 2012 in Sri Lanka^29^ who demonstrated that SF was more prevalent in the hill regions compared to the plains. A trend towards increased prevalence of ST in non-peri-forested areas and SF in peri-forested areas was observed. This is inferred as the univariate analysis suggested significant association whereas multivariate analysis was not significant. Nilgiris had the lowest sero-prevalence of ST which is explained by the fact this area had the lowest temperature, highest rainfall and highest elevation.

In summary, this is the first sero-prevalence study conducted in 5 different districts with different geography (Ecology). We used ELISA for determining IgG antibodies to scrub typhus, spotted fever, murine typhus and Q fever, whereas the preferred assay to detect exposure to these diseases is the IFA^52,53^. Further, samples from subjects staying at elevation between 1000 and 2000 MSL could not be performed.

Active sero-surveillance in the same and similar areas from different regions of India are needed validate the findings of this study and provide more information about the disease epidemiology. In addition, vector studies will also provide valuable information about vector-host dynamics and their correlation with ecological factors.

## Supplementary Information

Supplementary Information.

## Data Availability

The data generated and analyzed during the current study can be obtained on reasonable request from the corresponding author.

## References

[1] Luce-Fedrow A, Mullins K, Kostik AP, St John HK, Jiang J, Richards AL (2015). Strategies for detecting rickettsiae and diagnosing rickettsial diseases. Future Microbiol..

[2] Faruque LI, Zaman RU, Gurley ES, Massung RF, Alamgir ASM, Galloway RL (2017). Prevalence and clinical presentation of Rickettsia, Coxiella, Leptospira, Bartonella and chikungunya virus infections among hospital-based febrile patients from December 2008 to November 2009 in Bangladesh. BMC Infect. Dis..

[3] Snowden, J., Ladd, M. & King, K. C. Rickettsial Infection. In *StatPearls* (StatPearls Publishing, 2022) http://www.ncbi.nlm.nih.gov/books/NBK431127/ (Accessed 28 Mar 2022). 28613765

[4] Blanton LS (2013). Rickettsial infections in the tropics and in the traveler. Curr. Opin. Infect. Dis..

[5] Porter SR, Czaplicki G, Mainil J, Guattéo R, Saegerman C (2011). Q fever: Current state of knowledge and perspectives of research of a neglected zoonosis. Int. J. Microbiol..

[6] Rathi N, Rathi A (2010). Rickettsial infections: Indian perspective. Indian Pediatr..

[7] Xu G, Walker DH, Jupiter D, Melby PC, Arcari CM (2017). A review of the global epidemiology of scrub typhus. PLoS Negl. Trop. Dis..

[8] Sahni SK, Narra HP, Sahni A, Walker DH (2013). Recent molecular insights into rickettsial pathogenesis and immunity. Future Microbiol..

[9] Jiang J, Richards AL (2018). Scrub typhus: No longer restricted to the tsutsugamushi triangle. Trop. Med. Infect. Dis..

[10] Taylor AJ, Paris DH, Newton PN (2015). a systematic review of mortality from untreated scrub typhus (*Orientia** tsutsugamushi*). PLoS Negl. Trop. Dis..

[11] Abdad MY, Abou Abdallah R, Fournier P-E, Stenos J, Vasoo S (2018). A concise review of the epidemiology and diagnostics of rickettsioses: Rickettsia and *Orientia* spp. J. Clin. Microbiol..

[12] Kelly DJ, Richards AL, Temenak J, Strickman D, Dasch GA (2002). The past and present threat of rickettsial diseases to military medicine and international public health. Clin. Infect. Dis..

[13] Tshokey T, Stenos J, Durrheim DN, Eastwood K, Nguyen C, Graves SR (2017). Seroprevalence of rickettsial infections and Q fever in Bhutan. PLoS Negl. Trop. Dis..

[14] Khan SA, Bora T, Chattopadhyay S, Jiang J, Richards AL, Dutta P (2016). Seroepidemiology of rickettsial infections in Northeast India. Trans. R. Soc. Trop. Med. Hyg..

[15] Devamani CS, Schmidt W-P, Ariyoshi K, Anitha A, Kalaimani S, Prakash JAJ (2020). Risk factors for scrub typhus, murine typhus, and spotted fever seropositivity in urban areas, rural plains, and peri-forest hill villages in South India: A cross-sectional study. Am. J. Trop. Med. Hyg..

[16] Dhaka P, Malik SS, Yadav JP, Kumar M, Baranwal A, Barbuddhe SB (2019). Seroprevalence and molecular detection of coxiellosis among cattle and their human contacts in an organized dairy farm. J. Infect. Public Health..

[17] Yadav JP, Malik SVS, Dhaka P, Kumar A, Kumar M, Bhoomika S (2021). *Coxiella **burnetii* in cattle and their human contacts in a gaushala (cattle shelter) from India and its partial com1 gene sequence-based phylogenetic analysis. Anim. Biotechnol..

[18] Paulraj PS, Renu G, Ranganathan K, Leo VJ, Veeramanoharan R (2021). First seroprevalence report of scrub typhus from the tribal belts of the Nilgiris district, Tamil Nadu, India. Indian J. Med. Res..

[19] Lalrinkima H, Lalremruata R, Lalchhandama C, Khiangte L, Siamthara FH, Lalnunpuia C (2017). Scrub typhus in Mizoram, India. J. Vector Borne Dis..

[20] Kularatne SAM, Rajapakse RPVJ, Wickramasinghe WMRS, Nanayakkara DM, Budagoda SS, Weerakoon KGAD (2013). Rickettsioses in the central hills of Sri Lanka: Serological evidence of increasing burden of spotted fever group. Int. J. Infect. Dis..

[21] Agriculture.pdf. https://www.tn.gov.in/deptst/agriculture.pdf (Accessed 29 Mar 2022).

[22] Census of India Website: Office of the Registrar General & Census Commissioner, India. https://censusindia.gov.in/2011census/dchb/Tamilnadu.html (Accessed 29 Mar 2022).

[23] Tamil Nadu topographic map, elevation, relief. topographic-map.com. https://en-ca.topographic-map.com/maps/lggf/Tamil-Nadu/ (Accessed 29 Mar 2022).

[24] List of districts of Tamil Nadu https://www.census2011.co.in/census/state/districtlist/tamil+nadu.html (Accessed 29 Mar 2022).

[25] OpenEpi Menu https://www.openepi.com/Menu/OE_Menu.htm (Accessed 29 Mar 2022).

[26] Bharatmaps-GIS https://bharatmaps.gov.in/newversion/map.aspx (Accessed 29 Mar 2022).

[27] timeanddate.com https://www.timeanddate.com/ (Accessed 29 Mar 2022).

[28] Um J, Nam Y, Lim JN, Kim M, An Y, Hwang SH (2021). Seroprevalence of scrub typhus, murine typhus and spotted fever groups in North Korean refugees. Int. J. Infect. Dis..

[29] Kularatne SM, Edirisingha JS, Gawarammana IB, Urakami H, Chenchittikul M, Kaiho I (2003). Emerging rickettsial infections in Sri Lanka: The pattern in the hilly Central Province. Trop. Med. Int. Health..

[30] Bernabeu-Wittel M, del Toro MD, Nogueras MM, Muniain MA, Cardeñosa N, Márquez FJ (2006). Seroepidemiological study of *Rickettsia **felis*, *Rickettsia typhi*, and *Rickettsia conorii* infection among the population of southern Spain. Eur. J. Clin. Microbiol. Infect. Dis..

[31] Hsi T-E, Hsiao S-W, Minahan NT, Yen T-Y, de Assunção Carvalho AV, Raoult D (2020). Seroepidemiological and molecular investigation of spotted fever group rickettsiae and *Coxiella **burnetii* in Sao Tome Island: A One Health approach. Transbound. Emerg. Dis..

[32] Ming-yuan F, Walker DH, Shu-rong Y, Qing-huai L (1987). Epidemiology and ecology of rickettsial diseases in the People’s Republic of China. Rev. Infect. Dis..

[33] Londoño AF, Acevedo-Gutiérrez LY, Marín D, Contreras V, Díaz FJ, Valbuena G (2017). Human prevalence of the spotted fever group (SFG) rickettsiae in endemic zones of Northwestern Colombia. Ticks Tick Borne Dis..

[34] Mostafavi E, Molaeipoor L, Esmaeili S, Ghasemi A, Kamalizad M, YousefiBehzadi M (2019). Seroprevalence of Q fever among high-risk occupations in the Ilam province, the west of Iran. PLoS ONE.

[35] Forshey BM, Stewart A, Morrison AC, Gálvez H, Rocha C, Astete H (2010). Epidemiology of spotted fever group and typhus group rickettsial infection in the Amazon Basin of Peru. Am. J. Trop. Med. Hyg..

[36] Chang B-J, Kim S-J, Lee W-C, Lee M-J, Choe N-H (2018). Comparative study on the epidemiological trends and aspects of murine typhus in Korea in the last decade (2006–2015). J. Glob. Infect. Dis..

[37] Vallée J, Thaojaikong T, Moore CE, Phetsouvanh R, Richards AL, Souris M (2010). Contrasting spatial distribution and risk factors for past infection with scrub typhus and murine typhus in Vientiane City, Lao PDR. PLoS Negl. Trop. Dis..

[38] Mane A, Kamble S, Singh MK, Ratnaparakhi M, Nirmalkar A, Gangakhedkar R (2019). Seroprevalence of spotted fever group and typhus group rickettsiae in individuals with acute febrile illness from Gorakhpur, India. Int. J. Infect. Dis..

[39] Daniel SA, Manika K, Arvanmdou M, Antoniadis A (2002). Prevalence of *Rickettsia conorii* and *Rickettsia typhi* infections in the population of northern Greece. Am. J. Trop. Med. Hyg..

[40] da Costa PSG, Brigatte ME, Greco DB (2005). Antibodies to *Rickettsia **rickettsii*, *Rickettsia*
*typhi*, *Coxiella*
*burnetii*, *Bartonella*
*henselae*, *Bartonella*
*quintana*, and *Ehrlichia*
*chaffeensis* among healthy population in Minas Gerais, Brazil. Mem. Inst. Oswaldo Cruz..

[41] Tissot Dupont H, Raoult D, Brouqui P, Janbon F, Peyramond D, Weiller PJ (1992). Epidemiologic features and clinical presentation of acute Q fever in hospitalized patients: 323 French cases. Am. J. Med..

[42] Vikram K, Agarwala P, Bhargava A, Jain Y, Jagzape T, Wasnik P (2020). Scrub typhus and leptospirosis in rural and urban settings of central India: A preliminary evaluation. Trop. Doct..

[43] Bithu R, Kanodia V, Maheshwari RK (2014). Possibility of scrub typhus in fever of unknown origin (FUO) cases: An experience from Rajasthan. Indian J. Med. Microbiol..

[44] Hidalgo M, Montoya V, Martínez A, Mercado M, De la Ossa A, Vélez C (2013). Flea-borne rickettsioses in the north of Caldas province, Colombia. Vector Borne Zoonotic Dis..

[45] Weitzel T, Acosta-Jamett G, Jiang J, Martínez-Valdebenito C, Farris CM, Richards AL (2020). Human seroepidemiology of Rickettsia and Orientia species in Chile—A cross-sectional study in five regions. Ticks Tick Borne Dis..

[46] Gérardin P, Zemali N, Bactora M, Camuset G, Balleydier E, Pascalis H (2019). Seroprevalence of typhus group and spotted fever group Rickettsia exposures on Reunion island. BMC. Res. Notes.

[47] Obaidat MM, Malania L, Imnadze P, Roess AA, Bani Salman AE, Arner RJ (2019). Seroprevalence and risk factors for *Coxiella **burnetii* in Jordan. Am. J. Trop. Med. Hyg..

[48] Wangrangsimakul T, Elliott I, Nedsuwan S, Kumlert R, Hinjoy S, Chaisiri K, Day NPJ, Morand S (2020). The estimated burden of scrub typhus in Thailand from national surveillance data (2003–2018). PLoS Negl. Trop. Dis..

[49] Shah HA, Huxley P, Elmes J, Murray KA (2019). Agricultural land-uses consistently exacerbate infectious disease risks in Southeast Asia. Nat. Commun..

[50] Lu J, Liu Y, Ma X, Li M, Yang Z (2021). Impact of meteorological factors and southern oscillation index on scrub typhus incidence in Guangzhou, Southern China, 2006–2018. Front. Med..

[51] Lv Y, Guo X, Jin D, Song W, Peng P, Lin H (2021). Infestation and seasonal fluctuation of chigger mites on the Southeast Asian house rat (*Rattus **brunneusculus*) in southern Yunnan Province, China. Int. J. Parasitol. Parasites Wildl..

[52] Chaisiri K, Tanganuchitcharnchai A, Kritiyakan A, Thinphovong C, Tanita M, Morand S, Blacksell SD (2022). Risk factors analysis for neglected human rickettsioses in rural communities in Nan province, Thailand: A community-based observational study along a landscape gradient. PLoS Negl. Trop. Dis..

[53] De Boni L, Mall S, Msimang V, de Voux A, Rossouw J, Frean J (2022). Exposure of South African abattoir workers to *Coxiella **burnetii*. Trop. Med. Infect. Dis..

