# Supplementary material for: Geography and prevalence of rickettsial infections in Northern Tamil Nadu, India: a cross-sectional study
Source: Sci Rep. 2022 Dec 2;12:20798. doi: 10.1038/s41598-022-21191-7 (PMC9718799; doi:10.1038/s41598-022-21191-7)
Supplement: Supplementary file 1 — Supplementary Information. [file 41598_2022_21191_MOESM1_ESM.docx]

**Supplementary dataset:**

**Dataset 1:** Disease wise sero-prevalence in the study areas

| (n = 2565) | | Frequency | Percentage |
| --- | --- | --- | --- |
| Scrub Typhus | Sero-Positive | 359 | 14 |
|  | Sero-Negative | 2206 | 86 |
| Spotted Fever | Sero-Positive | 234 | 9.1 |
|  | Sero-Negative | 2331 | 90.9 |
| Murine Typhus | Sero-Positive | 94 | 3.7 |
|  | Sero-Negative | 2471 | 96.3 |
| Q Fever | Sero-Positive | 146 | 5.7 |
|  | Sero-Negative | 2419 | 94.3 |

**Dataset 2:** Proportion of individual Rickettsial infections among the sero-positive individuals

| Rickettsial Infection **(n = 832)** | | **Frequency** | **Percentage** |
| --- | --- | --- | --- |
| **Scrub Typhus** | Sero-Positive | 358 | 43.03 |
| **Spotted Fever** | Sero-Positive | 234 | 28.12 |
| **Murine Typhus** | Sero-Positive | 94 | 11.30 |
| **Q Fever** | Sero-Positive | 146 | 17.55 |

**Dataset 3: Rickettsial Sero-Positivity in the 8 areas surveyed in North Tamil Nadu**

**Dataset 4:** Association of Rickettsial infections with various demographic variables (univariate analysis)

| **Variables**  **(n = 2565)** | **Scrub Typhus** | | **Spotted Fever** | | **Q Fever** | | **Murine Typhus** | |
| --- | --- | --- | --- | --- | --- | --- | --- | --- |
|  | **Positive** | **p Value** | **Positive** | **p Value** | **Positive** | **p Value** | **Positive** | **p Value** |
| **Gender** | | | | | | | | |
| Female  (n = 1540) | 234 (15.2%) | 0.027* | 133  (8.6%) | 0.294 | 81 (5.3%) | 0.247 | 72  (4.7%) | 0.001* |
| Male  (n = 1025) | 124  (12.1%) |  | 101  (9.9%) |  | 65 (6.3%) |  | 22  (2.1%) |  |
| **Age Group** | | | | | | | | |
| 15 – 25 years  (n = 364) | 28 (7.7%) | <0.001* | 20 (5.5%) | 0.023* | 14 (3.8%) | 0.018* | 8 (2.2%) | 0.332 |
| 26 – 35 years  (n = 520) | 47 (9%) |  | 50 (9.6%) |  | 24 (4.6%) |  | 22 (4.2%) |  |
| 36 – 45 years  (n = 564) | 88 (15.6%) |  | 43 (7.6%) |  | 39 (6.9%) |  | 21 (3.7%) |  |
| 46 – 55 years  (n = 459) | 77 (16.8%) |  | 56 (12.2%) |  | 23  (5%) |  | 23  (5%) |  |
| 56 – 65 years  (n = 432) | 83 (19.2%) |  | 47 (10.9%) |  | 27 (6.3%) |  | 11 (2.5%) |  |
| Above 65 years  (n = 226) | 35 (15.5%) |  | 18  (8%) |  | 19 (8.4%) |  | 9  (4%) |  |
| **Place of residence** | | | | | | | | |
| Rural  (n = 1772) | 272  (15.3%) | 0.002* | 214  (12.1%) | <0.001* | 89  (5%) | 0.029* | 64  (3.6%) | 0.831 |
| Urban  (n = 793) | 86  (10.8%) |  | 20  (2.5%) |  | 57  (7.2%) |  | 30  (3.8%) |  |
| **Peri-forest** | | | | | | | | |
| Yes  (n = 1108) | 126  (11.4%) | 0.001* | 170  (15.3%) | <0.001* | 61  (5.5%) | 0.722 | 21  (1.9%) | <0.001* |
| No  (n = 1457) | 232  (15.9%) |  | 64  (4.4%) |  | 85  (5.8%) |  | 73  (5%) |  |
| **Districts** | | | | | | | | |
| Erode  (n = 406) | 9  (2.2%) | <0.001* | 0  (0%) | <0.001* | 26  (6.4%) | <0.001* | 9  (2.2%) | <0.001* |
| Jawadhi hills  (n = 205) | 30  (14.6%) |  | 42  (20.5%) |  | 2  (1%) |  | 0  (0%) |  |
| Kalrayan hills  (n = 331) | 53  (16%) |  | 99  (29.9%) |  | 38  (11.5%) |  | 8  (2.4%) |  |
| Nilgiri hills  (n = 345) | 7  (2%) |  | 18  (5.2%) |  | 15  (4.3%) |  | 7  (2%) |  |
| Palamathi hills  (n = 146) | 18  (12.3%) |  | 10  (6.8%) |  | 1  (0.7%) |  | 0  (0%) |  |
| Salem  (n = 181) | 19  (10.5%) |  | 3  (1.7%) |  | 21  (11.6%) |  | 1  (0.6%) |  |
| Tiruvannamalai  (n = 417) | 110  (26.4%) |  | 34  (8.2%) |  | 7  (1.7%) |  | 56  (13.4%) |  |
| Vellore  (n = 534) | 112  (21%) |  | 28  (5.2%) |  | 36  (6.7%) |  | 13  (2.4%) |  |

*p value <0.05

**Note**:

Areas with the lowest prevalence were taken as the reference for analysis of significance. Therefore, for scrub typhus, Nilgiris was the reference, for spotted fever and murine typhus, Salem, was the reference, whereas for Q fever it was Palamathi hills.
